# Supplementary material for: Smooth Interpolating Curves with Local Control and Monotone Alternating Curvature
Source: Comput Graph Forum. 2022 Oct 6;41(5):25–38. doi: 10.1111/cgf.14600 (PMC9827861; doi:10.1111/cgf.14600)
Supplement: Supplementary file 1 — Supplement Material [file CGF-41-25-s001.zip › Local-Smooth-Interpolating-MonoCurvature/extern/clothoids/docs/api-matlab/program_listing_file_ClothoidSplineG2.m.html]

Program Listing for File ClothoidSplineG2.m — Clothoids v2.0.9

### Navigation

- index
- toc
- Clothoids »
- Program Listing for File ClothoidSplineG2.m

# Program Listing for File ClothoidSplineG2.m¶

↰ Return to documentation for file (`ClothoidSplineG2.m`)

```
%>
%> Construct a piecewise clothoids \f$ \G(s) \f$ composed by
%> n clothoids that solve the G2 problem
%>
%> **match points**
%>
%> \f[ G(s_k) = \mathbf{p}_k \f]
%> \f[ \lim_{s\to s_k^+} G'(s) = \lim_{s\to s_k^-} G'(s) \f]
%> \f[ \lim_{s\to s_k^+} G''(s) = \lim_{s\to s_k^-} G''(s) \f]
%>
%> **Reference**
%>
%> The solution algorithm is described in
%>
%> - **E.Bertolazzi, M.Frego**, Interpolating clothoid splines with curvature continuity
%>   Mathematica Methods in the Applied Sciences, vol 41, N.4, pp. 1723-1737, 2018
%>
%> \rst
%>
%>   .. image:: ../../images/G2problem3arc.jpg
%>      :width: 80%
%>      :align: center
%>
%> \endrst
%>
classdef ClothoidSplineG2 < handle
  %% MATLAB class wrapper for the underlying C++ class
  properties (SetAccess = private, Hidden = true)
    objectHandle; % Handle to the underlying C++ class instance
    use_Ipopt;
    iter_opt;
    ipopt_check_gradient;
    isOctave;
  end

  methods (Hidden = true)
    % - - - - - - - - - - - - - - - - - - - - - - - - - - - - - - - - - - - - -
    function [ceq,jaceq] = nlsys( self, theta )
      ceq = ClothoidSplineG2MexWrapper( 'constraints', self.objectHandle, theta );
      jaceq = ClothoidSplineG2MexWrapper( 'jacobian', self.objectHandle, theta  );
    end
    % - - - - - - - - - - - - - - - - - - - - - - - - - - - - - - - - - - - - -
    function [c,ceq,jac,jaceq] = con( self, theta )
      c   = zeros(0,0);
      ceq = ClothoidSplineG2MexWrapper( 'constraints', self.objectHandle, theta );
      jac   = sparse(0,0);
      jaceq = ClothoidSplineG2MexWrapper( 'jacobian', self.objectHandle, theta  ).';
    end
    % - - - - - - - - - - - - - - - - - - - - - - - - - - - - - - - - - - - - -
    function [o,g] = obj( self, theta )
      o = ClothoidSplineG2MexWrapper( 'objective', self.objectHandle, theta );
      g = ClothoidSplineG2MexWrapper( 'gradient', self.objectHandle, theta );
    end
    % - - - - - - - - - - - - - - - - - - - - - - - - - - - - - - - - - - -
    %>
    %> Call C++ setup for the problem solver.
    %> Check that consecutive points are distinct
    %>
    function build( self, x, y )
      chk = diff(x).^2+diff(y).^2;
      [mi,idx1] = min(chk);
      [ma,idx2] = max(chk);
      if mi == 0 || mi < 1e-10*ma
        error( ...
          [ 'ClothoidSplineG2, build failed: minimum distance between ', ...
            'two consecutive points [min dist=%g, max dist=%g] is 0 or too small!\n', ...
            'index of points at minimum distance is %d\n'], mi, ma, idx1 ...
        );
      end
      ClothoidSplineG2MexWrapper( 'build', self.objectHandle, x, y );
    end
    % - - - - - - - - - - - - - - - - - - - - - - - - - - - - - - - - - - - - -
    function clots = build_internal( self, x, y, varargin )
      %
      % Compute guess angles
      %
      self.build( x, y );
      [ theta_guess, theta_min, theta_max ] = self.guess();
      [~,nc] = self.dims();

      if self.use_Ipopt

        options = {};

        options.ub = theta_max;
        options.lb = theta_min;

        % The constraint functions are bounded to zero
        options.cl = zeros(nc,1); %  constraints
        options.cu = zeros(nc,1);

        % Set the IPOPT options.
        options.ipopt.jac_d_constant      = 'no';
        options.ipopt.hessian_constant    = 'no';
        options.ipopt.mu_strategy         = 'adaptive';
        options.ipopt.max_iter            = 400;
        options.ipopt.tol                 = 1e-10;
        options.ipopt.derivative_test_tol = 1e-5;
        if self.ipopt_check_gradient
          options.ipopt.derivative_test = 'first-order';
        else
          options.ipopt.derivative_test = 'none';
        end
        %options.ipopt.derivative_test_perturbation = 1e-8;

        % The callback functions.
        funcs.objective         = @(theta) ClothoidSplineG2MexWrapper( 'objective', self.objectHandle, theta );
        funcs.constraints       = @(theta) ClothoidSplineG2MexWrapper( 'constraints', self.objectHandle, theta );
        funcs.gradient          = @(theta) ClothoidSplineG2MexWrapper( 'gradient', self.objectHandle, theta );
        funcs.jacobian          = @(theta) ClothoidSplineG2MexWrapper( 'jacobian', self.objectHandle, theta );
        funcs.jacobianstructure = @()      ClothoidSplineG2MexWrapper( 'jacobian_pattern', self.objectHandle );

        %options.ipopt.jacobian_approximation = 'finite-difference-values';
        options.ipopt.hessian_approximation  = 'limited-memory';
        %options.ipopt.limited_memory_update_type = 'bfgs'; % {bfgs}, sr1 = 6; % {6}
        %options.ipopt.limited_memory_update_type = 'sr1';
        options.ipopt.limited_memory_update_type = 'bfgs'; % {bfgs}, sr1 = 6; % {6}

        tic
        [theta, info] = ipopt(theta_guess,funcs,options);
        stats.elapsed = toc;
        info;
      else
        % 'interior-point'
        if self.isOctave
          options.TolX   = 1e-10;
          options.TolFun = 1e-20;
        else
          options = optimoptions(...
            'fmincon','Display',self.iter_opt, ...
            'CheckGradients',false, ...
            'FiniteDifferenceType','central', ...
            'Algorithm','sqp',...
            'SpecifyConstraintGradient',true,...
            'SpecifyObjectiveGradient',true,...
            'OptimalityTolerance',1e-20,...
            'ConstraintTolerance',1e-10 ...
          );
        end
        obj   = @(theta) self.obj(theta);
        con   = @(theta) self.con(theta);
        theta = fmincon(obj,theta_guess,[],[],[],[],theta_min,theta_max,con,options);
        %options = optimset(varargin{:});
        %[theta,resnorm,~,~,output,~,~] = lsqnonlin( @target, theta, [], [], options );
      end
      %
      % Compute spline parameters
      %
      clots = ClothoidList();
      N     = length(theta);
      clots.reserve(N-1);
      for j=1:N-1
        clots.push_back_G1( x(j), y(j), theta(j), x(j+1), y(j+1), theta(j+1) );
      end
    end
    % - - - - - - - - - - - - - - - - - - - - - - - - - - - - - - - - - - - - -
    function clots = build_internal2( self, x, y, varargin )
      %
      % Compute guess angles
      %
      self.build( x, y );
      [ theta_guess, ~, ~ ] = self.guess();
      % 'interior-point'
      if self.isOctave
        options.TolX = 1e-20;
      else
        options = optimoptions( ...
          'fsolve', 'Display', self.iter_opt, ...
          'CheckGradients', false, ...
          'FiniteDifferenceType', 'central', ...
          'Algorithm', 'levenberg-marquardt',...
          'SpecifyObjectiveGradient', true,...
          'OptimalityTolerance', 1e-20 ...
        );
      end
      obj = @(theta) self.nlsys(theta);
      [theta,~,exitflag,~] = fsolve(obj,theta_guess,options);
      if exitflag <= 0
        error('ClothoidSplineG2, fsolve failed exitflag = %d\n',exitflag);
      end
      %
      % Compute spline parameters
      %
      clots = ClothoidList();
      N     = length(theta);
      clots.reserve(N-1);
      for j=1:N-1
        clots.push_back_G1( x(j), y(j), theta(j), x(j+1), y(j+1), theta(j+1) );
      end
    end
  end

  methods
    % - - - - - - - - - - - - - - - - - - - - - - - - - - - - - - - - - - -
    function self = ClothoidSplineG2()
      self.objectHandle          = ClothoidSplineG2MexWrapper( 'new' );
      self.use_Ipopt             = false;
      self.iter_opt              = 'iter';
      self.ipopt_check_gradient  = false;
      self.isOctave              = exist('OCTAVE_VERSION', 'builtin') ~= 0;
    end
    % - - - - - - - - - - - - - - - - - - - - - - - - - - - - - - - - - - -
    function delete( self )
      ClothoidSplineG2MexWrapper( 'delete', self.objectHandle );
    end
    % - - - - - - - - - - - - - - - - - - - - - - - - - - - - - - - - - - -
    function verbose( self, yes )
      if yes
        self.iter_opt = 'iter';
      else
        self.iter_opt = 'none';
      end
    end
    % - - - - - - - - - - - - - - - - - - - - - - - - - - - - - - - - - - -
    function ipopt( self, yesno )
      self.use_Ipopt = yesno;
    end
    % - - - - - - - - - - - - - - - - - - - - - - - - - - - - - - - - - - -
    function ipopt_check( self, yesno )
      self.ipopt_check_gradient = yesno;
    end
    % - - - - - - - - - - - - - - - - - - - - - - - - - - - - - - - - - - -
    function [n,nc] = dims( self )
      [n,nc] = ClothoidSplineG2MexWrapper( 'dims', self.objectHandle );
    end
    % - - - - - - - - - - - - - - - - - - - - - - - - - - - - - - - - - - -
    function [ theta, theta_min, theta_max ] = guess( self )
      [ theta, theta_min, theta_max ] = ...
        ClothoidSplineG2MexWrapper( 'guess', self.objectHandle );
    end
    % - - - - - - - - - - - - - - - - - - - - - - - - - - - - - - - - - - -
    %>
    %> Compute the clothoid spline passing to the points (x(i),y(i))
    %> with initial angle theta0 (radiants) and final angle theta1 (radiants)
    %>
    function clots = buildP1( self, x, y, theta0, theta1 )
      ClothoidSplineG2MexWrapper( ...
        'target', self.objectHandle, 'P1', theta0, theta1 ...
      );
      clots = self.build_internal2( x, y );
    end
    % - - - - - - - - - - - - - - - - - - - - - - - - - - - - - - - - - - -
    %>
    %> Compute the clothoid spline passing to the points (x(i),y(i))
    %> with cyclic condition (tangent and curvature meet)
    %>
    %> - theta(1) = theta(end) mod 2*pi
    %> - kappa(1) = kappa(end)
    %>
    function clots = buildP2( self, x, y )
      ClothoidSplineG2MexWrapper( 'target', self.objectHandle, 'P2' );
      clots = self.build_internal2( x, y );
    end
    % - - - - - - - - - - - - - - - - - - - - - - - - - - - - - - - - - - -
    %>
    %> Compute the clothoid spline passing to the points (x(i),y(i))
    %> with assignede initial angle and survature
    %> NB: this target is not reccomended (its unstable), used only for debugging
    %>
    function clots = buildP3( self, x, y, theta0, kappa0 )
      ClothoidSplineG2MexWrapper( 'target', self.objectHandle, 'P3' );
      %
      % Compute spline parameters
      %
      clots = ClothoidList();
      N     = length(x);
      clots.reserve(N-1);

      c  = ClothoidCurve();
      ok = c.build_forward( x(1), y(1), theta0, kappa0, x(2), y(2) );
      if ok
        clots.push_back( c );
      else
        warning('buildP3 failed');
      end

      for j=3:N
        theta0 = c.thetaEnd();
        kappa0 = c.kappaEnd();
        ok = c.build_forward( x(j-1), y(j-1), theta0, kappa0, x(j), y(j) );
        if ok
          clots.push_back( c );
        else
          warning('buildP3 failed');
        end
      end
    end
    % - - - - - - - - - - - - - - - - - - - - - - - - - - - - - - - - - - -
    %>
    %> Compute the clothoid spline passing to the points \f$ (x_i,y_i)\f$
    %> and minimizing derivative of the curvature al the extrema
    %>
    %> minimize \f$ k'(0)^2 + k'(L)^2 \f$
    %>
    function clots = buildP4( self, x, y )
      ClothoidSplineG2MexWrapper( 'target', self.objectHandle, 'P4' );
      clots = self.build_internal( x, y );
    end
    % - - - - - - - - - - - - - - - - - - - - - - - - - - - - - - - - - - -
    %>
    %> Compute the clothoid spline passing to the points \f$ (x_i,y_i)\f$
    %> and minimizing the length of the first and last segment
    %>
    %> minimize \f$ (s_1-s_0)+(s_n - s_{n-1}) \f$
    %>
    function clots = buildP5( self, x, y )
      ClothoidSplineG2MexWrapper( 'target', self.objectHandle, 'P5' );
      clots = self.build_internal( x, y );
    end
    % - - - - - - - - - - - - - - - - - - - - - - - - - - - - - - - - - - -
    %>
    %> Compute the clothoid spline passing to the points \f$ (x_i,y_i)\f$
    %> and minimizing the total length of the spline
    %>
    %> \f$ L = s_n-s_0 \f$
    %>
    function clots = buildP6( self, x, y )
      ClothoidSplineG2MexWrapper( 'target', self.objectHandle, 'P6' );
      clots = self.build_internal( x, y );
    end
    % - - - - - - - - - - - - - - - - - - - - - - - - - - - - - - - - - - -
    %>
    %> Compute the clothoid spline passing to the points \f$ (x_i,y_i)\f$
    %> and minimizing the integral of the square of the curvature:
    %>
    %> minimize: \f$ \displaystyle \int_0^L k(s)^2 \mathrm{d}s \f$
    %>
    function clots = buildP7( self, x, y )
      ClothoidSplineG2MexWrapper( 'target', self.objectHandle, 'P7' );
      clots = self.build_internal( x, y );
    end
    % - - - - - - - - - - - - - - - - - - - - - - - - - - - - - - - - - - -
    %>
    %> Compute the clothoid spline passing to the points \f$ (x_i,y_i)\f$
    %> and minimizing the integral of the square of the curvature derivative:
    %>
    %> minimize:  \f$ \displaystyle \int_0^L k'(s)^2 \mathrm{d}s \f$
    %>
    function clots = buildP8( self, x, y )
      ClothoidSplineG2MexWrapper( 'target', self.objectHandle, 'P8' );
      clots = self.build_internal( x, y );
    end
    % - - - - - - - - - - - - - - - - - - - - - - - - - - - - - - - - - - -
    %>
    %> Compute the clothoid spline passing to the points \f$ (x_i,y_i)\f$
    %> and minimizing the integral of the jerk squared
    %>
    function clots = buildP9( self, x, y )
      ClothoidSplineG2MexWrapper( 'target', self.objectHandle, 'P9' );
      clots = self.build_internal( x, y );
    end
    %
  end
end
```

### Quick search

### Table of Contents

- Matlab Interface Manual
- C++ API
- MATLAB API

«
hide menu

menu
sidebar
»

### Navigation

- index
- toc
- Clothoids »
- Program Listing for File ClothoidSplineG2.m

© Copyright 2021, Enrico Bertolazzi and Marco Frego.
Created using Sphinx 4.2.0.
